# Collective Critical Care Ambulance: an innovative transportation of critical care patients by bus in COVID-19 pandemic response

**DOI:** 10.1186/s13049-021-00896-0

**Published:** 2021-06-04

**Authors:** Thierry Lentz, Charles Groizard, Abel Colomes, Anna Ozguler, Michel Baer, Thomas Loeb

**Affiliations:** SAMU des Hauts-de-Seine, Assistance Publique – Hôpitaux de Paris, Université Paris-Saclay, Hôpital Raymond Poincaré, 104, boulevard Raymond Poincaré, 92 380 Garches, France

**Keywords:** Emergency medical service, Critical care transport, Interhospital transfer of critically ill patients, Collective transport, Mass casualty incidents, Disaster

## Abstract

**Background:**

During the COVID-19 pandemic, as the number of available Intensive Care beds in France did not meet the needs, it appeared necessary to transfer a large number of patients from the most affected areas to the less ones. Mass transportation resources were deemed necessary. To achieve that goal, the concept of a Collective Critical Care Ambulance (CCCA) was proposed in the form of a long-distance bus re-designed and equipped to accommodate up to six intensive care patients and allow Advanced Life Support (ALS) techniques to be performed while en route.

**Methods:**

The expected benefit of the CCCA, when compared to ALS ambulances accommodating a single patient, was to reduce the resources requirements, in particular by a lower personnel headcount for several patients being transferred to the same destination. A foreseen prospect, comparing to other collective transportation vectors such as airplanes, was the door-to-door capability, minimalizing patients’ handovers for safety concerns and time efficiency.

With the project of a short-distance transfer of several Intensive Care Unit (ICU) patients together, the opportunity came to test the CCCA under real-life conditions and evaluate safely its technical feasibility and impact in time and resources saving, before it could be proposed for longer distances.

**Results:**

Four COVID-19 patients were transported over 37 km. All patients were intubated and under controlled ventilation. One of them was under Norepinephrine support. Mean loading time was 1 min 39 s. Transportation time was 29 min. At destination, the mean unloading time was 1 min 15 s. No serious adverse effect, in particular regarding hemodynamic instability or ventilation disorder, has been observed. No harmful incident has occurred.

**Conclusions:**

It was a very instructive test. Collective medical evacuation by bus for critically ill patients under controlled ventilation is suitable and easy to implement. Design, ALS equipment, power autonomy, safety and resources saving, open the way for carrying up to 6 ICU-patients over a long distance. The CCCA could bring a real added-value in an epidemic context and could also be helpful in many other events generating multiple victims such as an armed conflict, a terrorist attack or a natural disaster.

## Background

When the COVID-19 pandemic hit France in January 2020, it appeared quickly that the number of available Intensive Care Unit (ICU) beds would not meet the needs, notably in the *Grand Est* and the *Île-de-France* regions. Consequently, the Ministry of Health made the decision to transfer patients from saturated ICUs to less crowded ones by interhospital transfers to less affected regions. Numerous individual transfers were made by air, ground or sea transportation and collective transport innovative features appeared, such as 10 “hospital trains”, following a previously validated concept [1].

In this context, a long-distance bus turned into a Collective Critical Care Ambulance (CCCA) was considered for testing under real conditions.

The main objective of this trial was to validate a proof of concept for operating a long-distance bus as a CCCA, transporting critically ill patients requiring Advanced Life Support (ALS) techniques to be performed while en route, with a door-to-door capability reducing handover manoeuvres. The secondary objective was to validate the specifications of this CCCA.

## Methods

In an Emergency Medical Service (named SAMU) located in *Hauts-de-Seine* province, a working group dedicated to Exceptional Healthcare Situations (EHS Group) proceeded with the re-design of the interior space of a *Man™ Lion’s Coach* model, one of the largest buses currently in use.

### Project management

The EHS Group was entitled as the Project manager, by which decisions on main characteristics of the CCCA design were made. Because of the urgent need of mass patients transfers, issued specifications documents were reduced to a strict minimal. Administrative ambulance regulations waiving was granted by the *Prefecture des Hauts-de-Seine* authority. Having the CCCA ready to operate took no more than 2 days.

The Project engineering included: risk assessment, adaptation of ambulance standards to a bus, compliance of medical & technical procedures, quality assurance and ethical considerations. Special attention was given to easiness for entry, exit, as well as movements in the aisle, safeguarding from contamination and training.

### Conception

#### Equipment and design

The CCCA equipment included: stretchers, portable medical and monitoring equipment, oxygen and power supply, all securely fastened. Design works focussed on the optimization of the interior space and separation of “contaminated” versus “clean” areas.

**Fig. 1:**
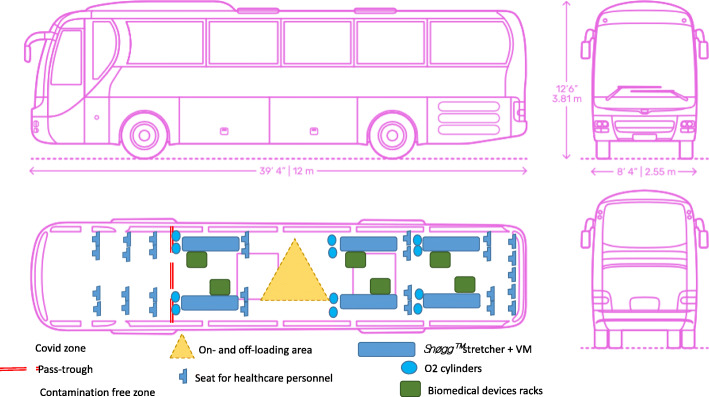
Sides view of CCCA and functional layout showing more available workspace compared with classical ambulance

**Fig. 2:**
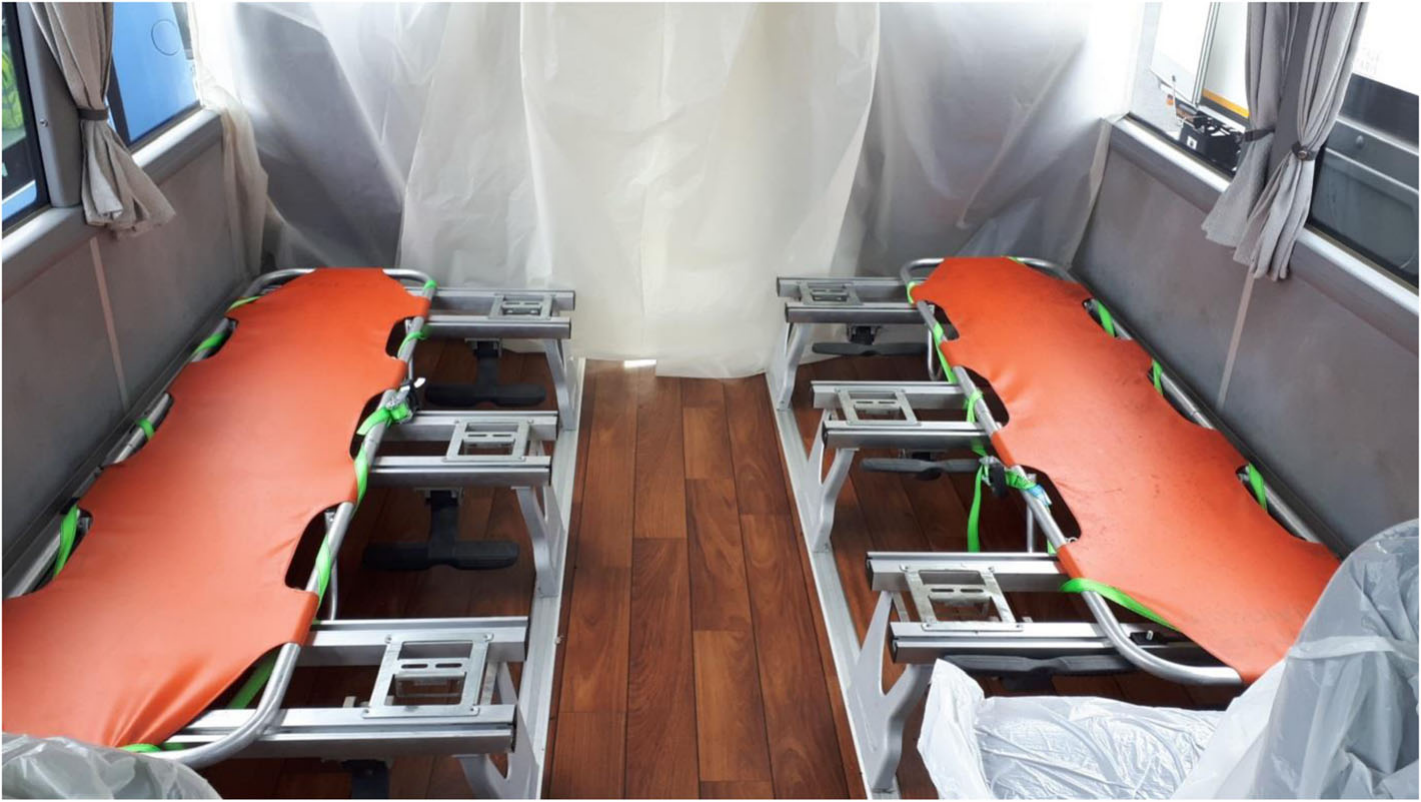
Two workstations in the “contaminated” area at the rear of the CCCA. The vertical vinyl film (seen in the back) creates a separation from a “clean” area located at the front of the bus, including the driver’s compartment and a rest area

Two distinct areas were separated by a vertical vinyl film: a large “contaminated” area at the rear, and a smaller “clean” area at the front including the driver’s compartment and a rest area. In the rear area, 6 groups of 4 seats each were removed, allowing for 6 stationary stretchers of the *Snøgg™* type to be rigged by retaining straps (Figs. 1 and 2). Air conditioning flow could not be completely shut down, and was reduced to the minimal.

The on-board equipment and healthcare personnel are listed in Table 1.

**Table 1:** Medical equipment & personnel dedicated to CCCA transport

Equipment	Count
Medical equipment for each workstation	
*Physio-Control Lifepak 15™* or *Lifepak 12™* multi-parametric monitor/defibrillator	1 per workstation
*Monnal T60™* or *Elisée 350™* ventilator	1 per workstation
Syringe pumps	4 per workstation
Suction unit	1 per workstation
Vacuum Mattress (VM)	1 per workstation
3 m^3^ Oxygen Cylinders (*p* = 200 bars)	2 per workstation
Power supply by transformer and batteries for Class II biomedical devices (stand-alone time = 60 h)	1 per workstation
Isothermal blanket	1 per workstation
Additional pooled resources available for the 6 workstations	
Large container for intensive care consumables and drugs	1
Large container for Personnel Protective Equipment (PPE) including: Disposable *Tyvek Classic Plus™* coveralls; FFP2 Masks; Gloves, shoe covers, etc.	1
*Abbott i-STAT System™* blood analyser	1
*Hemocue™ Hb 201 System* for hemoglobin testing	1
Spare equipment*	
Spare ventilator	1
1 m^3^ Oxygen Cylinders (p = 200 bars)	6
ALS ambulance stretcher trolleys	2
Healthcare personnel (CCCA medical team)	
Doctors (senior - resident)	2 (1–1)
Critical Care Nurses	4

*Spare equipment was boarded in the trunks (baggage compartments)

#### Training of healthcare personnel

Healthcare personnel operating the CCCA were all SAMU regular employees, working on a daily basis in ALS ambulances. Three of them were referent Chemical, Biological, Radiological and Nuclear (CBRN) instructors and all underwent regular training with personal protective equipment (PPE).

The driver was a volunteer employee of the Bus Company, with no additional training.

#### Patient triage

The exclusion rules were provided by the regional *SAMU zonal* authority: patients with FiO2 > 0.6, or Norepinephrine > 0.2 mg/h, or PEEP > 15 cm H2O were excluded.

### Assessment

The CCCA prototype first testing was made by a dedicated healthcare team already assigned among regular SAMU healthcare workers.

Main evaluation criteria were: time to finalize bus preparation, time data (loading, unloading, transportation), adverse unexpected events, patients and personnel safety.

HCW’s opinion on transfer conditions was collected in face to face interviews within the following days.

### Ethical consideration

All the transfers were made with the informed consent of the patients’ families. This survey obtained the MR-003 approval required by the National Council for Statistical Information (CNIL).

## Results – trial run description

On April 102,020, 4 COVID-19 patients from a hospital in the city of Corbeil (south of *Île-de-France* region) had to be transported to a Paris downtown station (*Gare d’Austerlitz*) prior to a transfer by a “hospital train” to Bordeaux (*Nouvelle-Aquitaine* region).

### Preparation

The day before, members of the EHS Group have been completing the preparation of the bus in 1h30min.

On D-day morning, CCCA went to Corbeil hospital and the healthcare team retrieved patients from ICUs at 6:05 am after getting dressed with PPE.

### Patients

The four patients, 2 female and 2 male, 58 years old average, were in a stable condition. They all suffered from COVID-19 related Acute Respiratory Distress Syndrome (ARDS), and had been hospitalised for 15 days in average at Corbeil hospital. They all were under continuous sedation and curarisation, intubated and mechanically ventilated with volume cycled assist-control mode. One of them was receiving 0.2 mg/h of Norepinephrine. They were monitored with Invasive Blood Pressure, EtCO2, SpO2 and ECG.

### Time

Patients’ loading and unloading were different from routine operations with ALS ambulances.. Mean measured loading time was 1 min 39 s. Mean measured unloading time was 1 min 15 s. Loading and unloading are considered from right outside the bus door to installation in the workstation and inversely.

The CCCA departed Corbeil at 7:32 am and arrived at *Gare d’Austerlitz* station at 8:01 am. after a 37 km drive.

### Transportation

Ventilation parameters and vasopressor agent infusion rate remained unchanged. Neither hemodynamic instability nor ventilation disorder has been observed. No harmful incident has occurred. Mutual support between healthcare personnel was perceived as a distinct advantage, when compared to lonely teams in a single ALS ambulance each.

Two technical problems occurred during the retrieval phase:

- A touchscreen of a ventilator appeared to be frozen, implying a switch to the spare ventilator and a maintenance manoeuvre;
- An ECG monitoring cable proved defective and a spare one had to be brought from the base hospital.

In addition, the CCCA team reported that the entrance of the bus was a narrow pass through a little staircase. Loading and unloading a patient “packaged” in a VM (handled from the sides) was not easy.

## Discussion

This experience was led in very good condition for patients, with no adverse effect during transport. It provided valuable information regarding collective evacuation by bus.

### Safety of transport

As stated above, some minor equipment dysfunctions were experienced, hinting at the need of additional spare peripheral items (connectors …) of every sensitive device.

There is no existing literature related to COVID-19 patients interhospital transportation so far. A 2016 review noticed that safety issues of interhospital transports were only recently the focus of some research [2]. Moreover, Droogh et al. [3] signalling that literature was addressing more often medical problems than technical ones, inventoried 55 types of such problems. Authors insisted as well on the ability of the transport team to resolve critical events, the rate of which was measured as concerning 16% of all patients in a 298 cases study [4], but a less worrisome 6.4% in another study involving 368 transports [5]. A 2020 evaluation of short-term mortality after 42,188 interhospital transports [6] advocates for transportation by ALS ambulance to minimize risks, as well as another study [7].

In summary, apart from having ample redundant spare items, the safety of such an interhospital transport relies essentially on the experience of assigned personnel. Specialized retrieval teams comprised of Emergency Medical Services (EMS) regular professionals (and not personnel whose work is restricted to the ICU) must be in charge, because of their specific knowledge of transport technical issues. The CCCA trial run personnel complied with this recommendation.

### Patients triage

When the aim of an interhospital transfer is to debottleneck a healthcare system, risks must be reduced as much as possible.

Strauch et al. evaluated short-term outcomes and mortality after interhospital transports by using the Sequential Organ Failure Assessment (SOFA) score [5]. An Experts’ Opinion [8] also recommended the SOFA score for pre-transport evaluation of patients, preferentially to the Simplified Acute Physiology Score (SAPS II) or the Acute Physiology and Chronic Health Evaluation Score (APACHE II). In this publication, patients were excluded if they met one of the following criteria: PaO2/FiO2 ratio < 100 with PEEP > 15 cmH2O, or mean arterial pressure < 60 mmHg despite adequate fluid therapy and vasoactive medication, or after cardiopulmonary resuscitation within 24 h prior to transport.

In the CCCA trial run, the *SAMU zonal* did provide the exclusion rules: patients with FiO2 > 0.6, or Norepinephrine > 0.2 mg/h, or PEEP > 15 cm H2O were excluded.

### Stretcher technique

Commonly, patients transported in ALS ambulance are “packaged” in a Vacuum Mattress (VM). Undoubtedly useful for immobilization of trauma patients [9], the VM is not so convenient when moving patients through a narrow corridor because it needs to be handled from the sides.

The entrance of a bus is such a narrow pass. To facilitate access on board, the VM could be associated with a Scoop Stretcher, the former being anchored on the latter. This way, the whole “package” could be handled from both extremities, making the operation smoother.

Consequently, a Scoop Stretcher must be added to the CCCA equipment.

### Handover of patients

SAMU of *Hauts-de-Seine* achieves annually 2200 interhospital transports. Its experience leads to emphasize the importance of a stage which interestingly was not stressed upon by the reviewed publications: the moment when devices connected to the patient in its ICU of origin, or in a given vector, have to be changed for other ones because of a handover. This coincides with the patient being moved from bed-to-stretcher (or stretcher-to-stretcher, or stretcher-to-bed) and generally also with a change of the healthcare team in charge.

The trial run showed that a considerable lapse of time was spent for this procedure, compared to the loading and unloading times.

#### Equipment changes

During these bed/stretcher change movements, accidental extubation or catheter disinsertion are likely incidents.

Other sensitive operations during handover of patients are:

- Ventilator change with parameters potentially differing from one model to another;
- Invasive blood pressure to be reinitialised (for zero-level);
- Syringe pumps to be changed with the risk of infusion irregularities;
- And a more or less brutal relocation of the patient onto a new surface.

Thus, limiting these equipment changes in interhospital transports is a safety matter, advocating for door-to-door capability of the vector.

#### Crew changes

When the responsibility of a critically ill patient is being transferred from a healthcare team to another, a comprehensive data transmission is mandatory [10]. A 2019 qualitative study of interhospital transports pointed out the discrepancy between the time spent on the road and the total time of the mission, explaining this by how time-consuming it was to prepare for a transport, collect information, etc. [11]

Again, limiting these crew changes in interhospital transfers pleads for door-to-door capability of the vector.

### Door-to door capability

In their very comprehensive synthesis about methods and issues of interhospital individual transports of patients with ARDS, Jahn et al. [12] outlined the need for not losing time, particularly because of the inability to escalate the therapeutic while en route. They notably insisted on the two necessary intermediate legs of a transport by fixed-wings aircraft, i.e.: from airport to hospital and from hospital to airport, by comparison with helicopters which may land at the hospital itself.

Considering collective transportation vectors, fixed-wings aircrafts and trains require these two intermediate legs between airport or station and hospitals, implying 4 crew changes/equipment changes.

For short distances, because of its door-to-door capability, a bus could be a suitable alternative to reduce these crew changes/equipment changes from 4 to 2. The CCCA accommodating only up to 6 patients, a balanced study between vectors capacities and transport length has to be made on a case-by-case basis.

### Safeguarding from contamination

Lockhart et al. [13] highlighted the risks associated with COVID-19 PPE doffing process, when healthcare workers “let their guard down”.

During the “hospital train” trip subsequent to the first leg transport by CCCA, CBRN referent instructors set up a decontamination/doffing pass-through at the bottom of the staircase to the upper deck of the wagon (considered as a “safe area”), the “contaminated area” being by definition the lower deck. This allowed healthcare workers to have a rest during an extended period of work under PPE. This pass-through has to be added to the CCCA configuration for future long-distance transports.

Finally, the air-conditioning system (A/C) was set at the lowest pace because it could not be shut down, the quality of A/C filters being unknown. Healthcare workers being under PPE, this was likely inconsequential for a short trip. For long-distance transports or patients under non-invasive ventilation (NIV), completely shutting down A/C or considering high efficiency filters will be necessary.

### Military vs civilian concept for collective evacuations

The French Army’s Health Service has been developing since 2006 a collective strategic air medical evacuation capability known as *Morphée* (*Module de Réanimation Pour Haute Élongation d’Évacuation*) [14]. Aboard an *Airbus A330 MRTT Phénix*, it may accommodate 6 to 12 patients over a 10,000 km range.

There is a doctrinal difference between these military collective evacuation resources and the CCCA civilian solution: whereas the civilian vectors are a one-off and engineered for a specific situation, the military have been developing a medical support concept based on early strategic aeromedical evacuation (MEDEVAC) [15]. This strategic MEDEVAC itself is the third leg of a survival chain from the point of injury to homeland [16]. Obviously, these military evacuations have to be performed for patients whose clinical status is not in a stable condition and en route stabilization capabilities are mandatory [17], offering a distinct survival advantage [18].

Civilian-organised collective evacuations cannot address the wartime tactical or strategic situations, hence must be devoted to stabilized (triaged) patients, even if under critical care support, patients eligibility through an appropriate application of risk scores [8] must be evaluated.

During civilian disasters, it is conceivable that stabilized patients may be evacuated by a collective civilian-organised transport such as a CCCA, from the scene to remote hospitals. Another conceivable circumstance is a sudden important need of a sparse specific capability such as burn intensive care beds.

## Conclusion

This first test of interhospital transport by a CCCA along a short distance with 4 patients was conducted in very good conditions, with no adverse effect or harmful incident. The experience brought a lot of information regarding safety, capabilities and time efficiency.

Compared to ALS ambulance accommodating a one only patient, the CCCA reduced the resources requirements by a lower personnel headcount, with more space, and improved indoor comfort conditions for staff. A foreseen prospect was a door-to-door capability minimalizing patients’ handovers for safety concerns and time efficiency. The very short time allowed to organize, rule and conceptualize the CCCA characterizes the emergency context during this COVID-19 pandemic.

The next step would be to test the CCCA on a long-distance transport, in order to evaluate cost-benefit aspects and total transport delays reduction.

## Data Availability

All data generated or analysed during this study are included in this published article.

## Abbreviations

A/C: Air-Conditioning System
ALS: Advanced Life Support
APACHE II: Acute Physiology and Chronic Health Evaluation Score
ARDS: Acute Respiratory Distress Syndrome
CCCA: Collective Critical Care Ambulance
CBRN: Chemical, Biological, Radiological and Nuclear
CNIL: National Council for Statistical Information
COVID19: Corona Virus Disease 2019
ECG: Electrocardiogram
EHS: Exceptional Healthcare Situations
EMS: Emergency Medical Services
Etco2: End-Tidal Carbon Dioxide
Fi02: Fraction of Inspired Oxygen
HCW: Healthcare Workers
ICU: Intensive Care Unit
MEDEVAC: Aeromedical Evacuation
Morphée: Module de Réanimation pour Haute Élongation d’Évacuation: Collective Strategic Air Medical Evacuation Capability developed by the French Army
MR-003: Méthodologie de Référence 003
NIV: Non-invasive ventilation
Pao2: Partial Pressure of Oxygen in Arterial Blood
PEEP: Positive End Expiratory Pressure
PPE: Personnel Protective Equipment
SAMU: Service d’Aide Médicale Urgente
SAPS II: Simplified Acute Physiology Score
SOFA: Sequential Organ Failure Assessment
Spo2: Saturation of Hemoglobin with Oxygen
VM: Vacuum Mattress
